# Pleiotrophin expression and role in physiological angiogenesis *in vivo*: potential involvement of nucleolin

**DOI:** 10.1186/2045-824X-4-4

**Published:** 2012-03-16

**Authors:** Marina Koutsioumpa, Georgia Drosou, Constantinos Mikelis, Katerina Theochari, Dionussios Vourtsis, Panagiotis Katsoris, Efstathia Giannopoulou, Jose Courty, Christos Petrou, Vassiliki Magafa, Paul Cordopatis, Evangelia Papadimitriou

**Affiliations:** 1 Laboratory of Molecular Pharmacology, Department of Pharmacy, University of Patras, Patras GR 26504, Greece; 2 Division of Genetics, Cell & Developmental Biology, Department of Biology, University of Patras, Patras, Greece; 3 Laboratoire CRRET, Université Paris Est Créteil, Val de Marne, 61 avenue du Général de Gaulle, 94010 Créteil, Cedex, France; 4 Laboratory of Pharmacognocy and Chemistry of Natural Products, Department of Pharmacy, University of Patras, Athens, Greece; 5 Oral and Pharyngeal Cancer Branch, National Institute of Dental and Craniofacial Research, National Institutes of Health, 30 Convent Drive, Building 30, Room 203, Bethesda, MD 20892-4340, USA; 6 Clinical Oncology Laboratory, Division of Oncology, Department of Medicine, University Hospital of Patras, Patras Medical School, 26504 Rio, Greece

**Keywords:** angiogenesis, endothelial cells, migration, nucleolin, pleiotrophin, receptor protein tyrosine phosphatase

## Abstract

**Background:**

Pleiotrophin (PTN) is a heparin-binding growth factor with significant role(s) in tumour growth and angiogenesis. Although implication of endogenous PTN has been studied in several *in vivo *models of tumour angiogenesis, its role in physiological angiogenesis has not been addressed. In the present work, we studied expression and functional significance of endogenous PTN during angiogenesis in the chicken embryo chorioallantoic membrane (CAM).

**Methods:**

Using molecular, cellular and biochemical assays, we studied the expression pattern of PTN in CAM and human endothelial cells and its possible interaction with nucleolin (NCL). CAM cells were transfected with a pCDNA3.1 vector, empty (PC) or containing full length cDNA for PTN in antisense orientation (AS-PTN). Angiogenesis was estimated by measuring total vessel length. *In vitro*, human endothelial cells migration was studied by using a transwell assay, and down-regulation of NCL was performed by using a proper siRNA.

**Results:**

Endogenous PTN mRNA and protein levels, as well as protein levels of its receptor protein tyrosine phosphatase beta/zeta (RPTPβ/ζ) were maximal at early stages, when CAM angiogenesis is active. Application of AS-PTN onto CAM at days of active angiogenesis was not toxic to the tissue and led to dose-dependent decreased expression of endogenous PTN, ERK1/2 activity and angiogenesis. Interestingly, endogenous PTN was also immunolocalized at the endothelial cell nucleus, possibly through interaction with NCL, a protein that has a significant role in the nuclear translocation of many proteins. Down-regulation of NCL by siRNA in human endothelial cells significantly decreased nuclear PTN, verifying this hypothesis. Moreover, it led to abolishment of PTN-induced endothelial cell migration, suggesting, for the first time, that PTN-NCL interaction has a functional significance.

**Conclusions:**

Expression of endogenous PTN correlates with and seems to be involved in angiogenesis of the chicken embryo CAM. Our data suggest that NCL may have a role, increasing the number of growth factors whose angiogenic/tumorigenic activities are mediated by NCL.

## Background

Pleiotrophin (PTN) is an 18 kDa secreted protein with high affinity for heparin. It is highly conserved among species and together with midkine, belongs to a family of heparin-binding growth factors with many similar biological activities. It was initially purified from bovine uterus and neonatal rat brain, and its expression has been detected increased in several developing tissues, such as the nervous system, deciduas basalis and mammary gland, bone and cartilage, liver, kidney, lung, the epithelial ridge of the cochlea, retinas and corneas. Although its functional role has not been always elucidated, it displays important functions in growth and differentiation processes, such as neurite outgrowth and synaptic plasticity, fertility, development and regeneration of liver, auditory function, would healing and adipogenesis. The best characterized functions of PTN up to date are those concerning its role(s) in the nervous system, as well as its involvement in tumour growth. The latter was initially supported by the fact that PTN has been detected in various human carcinomas, such as meningiomas, neuroblastomas, diffuse astrocytomas, glioblastomas, melanomas, multiple myeloma, prostate cancer, cancer of the pancreas, breast cancer, small cell lung cancer, malignant tumor of the testis, solid paediatric tumours, uterine cervical cancer and leiomyomas, while it has been also detected in serum of patients with breast, colon, pancreas, lung cancers and multiple myeloma. It is also constitutively expressed in cell lines derived from different types of tumours and is involved in tumour growth and metastasis in several experimental models. When PTN expression is up-regulated in normal cells the latter acquire a more malignant phenotype, while down-regulation of PTN expression decreases tumour cell proliferation and invasion *in vitro*, as well as tumour growth, metastases and angiogenesis *in vivo*, indicating a possible implication of PTN in the blood vessel network formation of solid tumours reviewed in [1,2]. Besides a role in tumour angiogenesis, we have previously shown that exogenously administered PTN induces angiogenesis in several *in vitro *models of angiogenesis [3-5] and *in vivo*, in the chicken embryo chorioallantoic membrane (CAM) [3]. Although the role of PTN has been studied in several *in vivo *models of tumour growth, there are no *in vivo *studies on the role of endogenous PTN in physiological angiogenesis.

PTN acts through several cell surface receptors, such as N-syndecan, anaplastic lymphoma kinase (ALK), receptor protein tyrosine phosphatase beta/zeta (RPTPβ/ζ) and α_ν_β_3 _integrin. Although ALK has been implicated in actions of PTN related to tumour growth, the best characterized receptor up to date, implicated in the tumour promoting activities of PTN is RPTPβ/ζ reviewed in [1,2]. We have previously shown that RPTPβ/ζ is required for the stimulatory effects of PTN on human endothelial cell migration and tube formation on matrigel *in vitro *[4] but only when α_ν_β_3 _integrin is also expressed [5]. Expression of integrin α_ν_β_3 _is low in quiescent and high in angiogenic endothelial cells [6]; in CAM endothelial cells, α_ν_β_3 _is expressed during the stages of new blood vessel formation [7]. Nucleolin (NCL) is also considered a low affinity receptor for PTN [8], although it has not been connected to any of PTN's biological activities. NCL is a multifunctional nucleolar protein that among other functions, acts as a shuttling protein between cytoplasm and nucleus [9]. NCL has been shown to import midkine into the nuclear fraction [10]. Based on the similarity of the two molecules, it has been hypothesized that NCL may also import PTN into the nuclear fraction [10], although this has never been shown up to date.

In the present study, we investigated the expression and the possible role of endogenous PTN and its receptors in the *in vivo *angiogenesis model of the chicken embryo CAM. Expression of endogenous PTN and its receptor RPTPβ/ζ correlates with angiogenesis of the chicken embryo CAM. PTN seems to be involved in CAM angiogenesis and NCL may have a role in the angiogenic functions of PTN both *in vivo *and *in vitro*.

## Methods

### Antibodies, oligonucleotides, and reagents

Cell culture reagents were from BiochromKG (Seromed, Germany). Human recombinant PTN was from Peprotech Inc (Rocky Hill, NJ, USA) or was prepared as previously described [3]. Monoclonal antibody against NCL, affinity purified rabbit polyclonal antibody against PTN (used for immunoprecipitations and immunohistochemistry of CAM paraffin sections) and goat polyclonal antibody against RPTPβ/ζ were from Santa Cruz Biotechnology Inc (Santa Cruz, CA, USA). Monoclonal antibody against PTN (used only in human cells) was from Abnova (Taipei City, Taiwan), goat polyclonal antibody against PTN was from R&D Systems (Minneapolis, MN, USA), antibody against phospho-ERK1/2 on Thr^202^-Tyr^204 ^was from Cell Signalling (Danvers, MA, USA), antibody against ERK1/2 was from Upstate Biotechnology (Lake Placid, NY, USA), antibody against Prox-1 was from Axxora (San Diego, CA, USA), antibodies against β-actin and α_ν_β_3 _were from Chemicon (Temecula, CA, USA) and monoclonal antibody against RPTPβ/ζ was from BD Transduction Laboratories (San Diego, CA, USA). Protein A and G agarose beads were purchased from Merck (Whitehouse Station, NJ, USA). DNA oligonucleotide primers for chicken PTN and GAPDH and RNA oligonucleotide primers for NCL were obtained from VBC Biotech Services (Vienna, Austria). Double-stranded negative control siRNA was obtained from Ambion (Austin, TX, USA) and the transfection reagents Jet-PEI and JetSI-ENDO were from Polyplus Transfection (Illkirch, France). Alexa secondary antibodies were from Molecular Probes (Carlsbad, CA, USA). Rabbit polyclonal antibody against NCL (used only in immunofluorescent studies), human IgG and all secondary horseradish peroxidase-conjugated antibodies were purchased from Sigma (St. Louis, MO, USA). Draq5 was from Biostatus Limited (Leicestershire, United Kingdom). The 9-fluorenylmethoxycarbonyl (Fmoc)-protected amino acids, Wang resin and peptide reagents were purchased from CBL (Patras, Greece), Bachem (Bubendorf, Switzerland) and Novabiochem (Läufelfingen, Switzerland). All other reagents, unless mentioned below, were purchased from Sigma or Applichem (Darmstadt, Germany).

### CAM assay

The *in vivo *chicken embryo CAM angiogenesis assay was used, as previously described [3]. Leghorn fertilized eggs (Pindos, Ioannina, Greece) were incubated for 4 days at 37°C, when a window was opened on the egg shell, exposing the chorioallantoic membrane. The window was covered with tape and the eggs were returned to the incubator. Different amounts of pCDNA3.1 alone or pCDNA3.1 carrying full length cDNA for PTN in antisense orientation (AS-PTN) [11], were diluted in a final volume of 50 μl of phosphate buffered saline pH 7.4 (PBS) containing jet-PEI (N/P = 5 ratio) and were applied at the 9^th ^day of embryo development on an area of 1 cm^2 ^of the CAM, restricted by a plastic ring. Forty eight hours after treatment and subsequent incubation at 37°C, CAMs were fixed in situ, excised from the eggs, placed on slides and left to air-dry. Pictures were taken through a stereoscope equipped with a digital camera and the total length of the vessels was measured, as previously described [3]. Assays were carried out three times and each experiment contained 10-20 eggs per data point.

For the biochemical studies, plasmids were applied on the CAM as described above, and after 24 h of incubation at 37°C, the CAMs were excised from the eggs, cut in pieces, washed three times in PBS and stored at -80°C until used [12]. Assays were carried out three times and each experiment contained 5-10 eggs per data point.

### Haematoxylin-eosin staining and immunohistochemistry of CAM paraffin sections

Tissues from various developmental stages were excised from the eggs, washed in PBS, fixed in saline-buffered formalin and embedded in paraffin. Sections were cut at 5 μm thickness and placed on positively charged glass slides. After rehydration, the tissue sections were stained with standard haematoxylin-eosin staining or processed for immunohistochemistry. In the latter case, endogenous peroxidase was blocked with 3% H_2_O_2 _for 30 min in a dark chamber at room temperature. Tissue sections were then incubated with blocking agent (Kwik Kits, Immunon Immunohistochemicals, Lipshaw, USA) for 15 min at room temperature to prevent non specific binding of antibodies, followed by incubation with 5 μg/ml of affinity purified rabbit anti-PTN IgG in Tris-buffered saline (TBS), pH 7.4, with 0.05% Tween (TBS-T) containing 2% bovine serum albumin (BSA) for 1 h at 37°C. After 3 washes of 2 min each in TBS-T, a second 30 min incubation at room temperature, using a horseradish peroxidase conjugated goat anti-rabbit IgG was performed at a dilution of 1:5,000 in TBS-T containing 2% BSA. After three washes of 2 min each in TBS, detection of PTN was performed by DAB staining. Sections were mounted in mounting fluid, viewed in a Zeiss microscope and photographed using a digital camera [12].

### Cell culture

Human umbilical vein endothelial cells (HUVEC) used in the present study were isolated from human umbilical cords and cultured as previously described [3]. HUVEC were grown as monolayers in medium M199 that was supplemented with 15% fetal bovine serum (FBS), 150 μg/ml endothelial cell growth supplement, 5 U/ml heparin sodium, 100 U/ml penicillin, 100 μg/ml streptomycin, 50 μg/ml gentamycin and 2.5 μg/ml amphotericin B and used at passages 2-3. U87MG cells (ATCC) were grown routinely in Dulbecco's modified Eagle medium (DMEM)/Ham's F12 medium supplemented with 10% FBS, 100 IU/ml penicillin, 100 μg/ml streptomycin, 50 μg/ml gentamycin, and 2.5 μg/ml amphotericin B. Cultures were maintained at 37°C, 5% CO_2_, and 100% humidity. When cells reached 70-80% confluence, they were lysed for immunoprecipitation experiments or fixed for immunofluorescent studies.

### Migration assay

Migration assays were performed as described previously [3,5] in 24-well microchemotaxis chambers (Corning, Inc., Lowell, MA, USA) using uncoated polycarbonate membranes with 8-μm pores. Serum-starved cells were harvested and resuspended at a concentration of 10^5 ^cells/0.1 ml in serum-free medium containing 0.25% BSA. The bottom chamber was filled with 0.6 ml of serum-free medium containing 0.25% BSA and the tested substances. The upper chamber was loaded with 0.1 ml of serum-free medium containing 10^5 ^cells and incubated for 4 h at 37°C. After completion of the incubation, the filters were fixed and stained with 0.33% toluidine blue solution. The cells that migrated through the filter were quantified by counting the entire area of each filter, using a grid and an Optech microscope at X20 (Optech Microscope Services Ltd., Thame, UK).

### Western blot analysis

CAMs from various developmental stages or after treatment with the plasmids were homogenized using a glass-glass homogenizer in 20 mM Hepes, pH 7.4, containing 2 M NaCl supplemented with 1 mM phenylmethylsulfonyl fluoride (PMSF), 5 mM EDTA and 1 μg/ml aprotinin. The homogenate was centrifuged at 10,000 x g for 20 min at 4°C. Equal amounts (100 μg) of total protein from CAM lysates or the immunoprecipitated samples, as described below, were analysed by SDS-PAGE and transferred to Immobilon P membranes. Blocking was performed by incubating the membranes with TBS-T containing 3% BSA in the case of PTN, RPTPβ/ζ, PROX-1, phosphorylated ERK1/2 (pERK1/2) and total ERK1/2 (tERK1/2) and 5% non-fat dry milk in the case of NCL and actin. Membranes were incubated with primary antibodies for 16 h at 4°C under continuous agitation, washed 3 times with TBS-T, and incubated with secondary antibodies for 1 h at room temperature. Membranes were finally washed and detection of immunoreactive bands was performed using the ECL detection kit (Pierce), according to the manufacturer's instructions. Blots for PTN, NCL and PROX-1, where appropriate, were stripped and subjected to subsequent Western blotting for actin. Blots for pERK1/2 were stripped and subjected to subsequent Western blotting for total ERK1/2. The protein amounts that corresponded to each immunoreactive band were quantified from digital images of gels, using the ImagePC image analysis software (Scion Corporation, Frederick, MD) [5,12].

### Immunoprecipitation assays

CAMs or cells were homogenised or lysed, respectively, in PBS containing 1% Triton X-100, 0.1% SDS, 20 nM sodium orthovanadate, 1 μg/ml aprotinin, 1 mM phenylmethylsulfonyl fluoride and 5 mM EDTA. Homogenates or lysates were centrifuged at 20,000 g for 30 min at 4°C. Three mg of total protein were transferred to new eppendorf tubes and incubated with primary antibodies for 16 h at 4°C under continuous agitation. Protein A- and protein G-agarose beads were added and samples were further incubated for 2 h at 4°C. Beads and bound proteins were collected by centrifugation and washed twice with ice-cold PBS [5]. The pellet was resuspended in 50 μl SDS loading buffer, heated to 95-100°C for 5 min, centrifuged and analyzed by Western blot analysis as described above.

### Reverse transcriptase-polymerase chain reaction (RT-PCR) for PTN

Total RNA was extracted from CAMs of various developmental stages using the Nucleospin RNA II kit (Macherey-Nagel, Germany), according to the manufacturer's instructions. Primers used for the detection of PTN mRNA were designed according to the chicken sequence (Accession number BI394859) and were: 5'-AGA GAA ACC AGA GAA AAA GG-3' (sense) and 5'-CAG TCA GCA TTA TGA AGA GC-3' (antisense), yielding a product of 288 bp. The reporter gene was the chicken glyceraldehyde-3-phosphate dehydrogenase (GAPDH) and the primers used were: 5'-ACG GAT TTG GCC GTA TTG GC-3' (sense) and 5'-GCA GGA TGC GAA ACT GAG CG-3' (antisense) [13]. The RT-PCR reactions for PTN and GAPDH were performed in a single step, using the Access RT-PCR system (Promega) under the following conditions: The reverse transcriptase reaction was performed by AMV-RT for 1 h at 48°C. After an initial denaturation step for 2 min at 94°C, 30 cycles of amplification (94°C for 1 min, 57°C for 40 sec and 68°C for 1.5 min for GAPDH and 94°C for 1 min, 55°C for 40 sec and 68°C for 1.5 min for PTN) were performed and ended with a final DNA synthesis step at 68°C for 7 min. In all cases, PCRs were not in the saturating phase (data not shown). DNA contamination was excluded by performing PCR reactions in the absence of the reverse transcription step. The RT-PCR products were subjected to electrophoresis on 2% agarose gels containing 0.5 μg/ml ethidium bromide and photographed using a digital camera. The bands were quantified (area and intensity) using Image PC image analysis software and the ratios PTN/GAPDH of electrophoretic band values represent the relative expression of *ptn *gene at different days of embryo development.

### RNA interference

The short interfering RNA against NCL was sense: 5'-GGAAGGUCAGCAGUCUUCCAUGAGA-3' and antisense: 5'-UCUCAUGGAAGACUGCUGACCUUCC-3' [14]. HUVEC were grown to a confluence of 50% in medium without antibiotics. Transfection was performed in serum-containing medium for 4 h using annealed RNA for NCL at the concentration of 50 nM and jetSI-ENDO as transfection reagent. Cells were incubated for another 24 h in serum-containing medium and lysed in order to evaluate transfection efficiency by Western blot analysis, or fixed for immunofluorescent studies. Double-stranded negative control siRNA from Ambion (catalogue # AM4635) was used in all assays.

### Immunofluorescence

For immunofluorescent studies, cells were fixed by 4% paraformaldehyde for 10 min. After being washed 3 times with PBS, the cells were blocked with PBS containing 3% BSA and 10% FBS for 1 h at room temperature. The cells were stained with primary antibodies against NCL (1:1,000) and PTN (1:500). Nuclei were stained with Draq5 (final concentration 3.3 μM). Fluorescent secondary antibodies were used at the concentration of 1:500, and the cells were mounted with Mowiol 4-88 (Calbiochem, San Diego, CA, USA) and visualized at 21°C with Leica SP5 (X63 objective with a numerical aperture of 1.4; Leica Microsystems, Wetzlar, Germany) confocal microscope.

### Subcellular fractionation

Subcellular fractions of U87MG cells comprising cytosolic, nuclear and cell membrane extracts were prepared as follows [15]: Cell monolayers in 100-mm plates were washed extensively with PBS before being scraped and pelleted. Washed cells (30 × 10^9^) were then disrupted in a hypotonic solution (10 mM Hepes, pH 6.9, 10 mM KCl, 2 mM MgCl_2_, 1000 units/ml aprotinin, 0.1 mM PMSF) on ice. Nuclei were pelleted at 400 *g *for 5 min and washed twice in PBS before extraction in the lysis buffer (10 mM Tris-HCl, pH 7.6, 400 mM NaCl, 1 mM EDTA, 1000 units/ml aprotinin, 0.1 mM PMSF, and 1% Triton X-100). The lysate was centrifuged at 12,000 *g *for 10 min and the supernatant was referred to as the nuclear fraction. The supernatant obtained after pelleting intact nuclei was further centrifuged at 14,000 *g *for 30 min and the supernatant corresponding to the cytosolic fraction was recovered, while the pellet was resuspended in lysis buffer containing 150 mM instead of 400 mM NaCl. This latter suspension was centrifuged at 14,000 *g *for 30 min to separate the cytoskeletal (the pellet) and membrane (supernatant) fractions. Equivalent total protein amounts of each fraction corresponding to nuclei, cytosol, and membrane were immunoprecipitated for PTN and then analyzed by Western blot analysis for PTN or NCL.

### Synthesis of the 5(KPR)TASP peptide

5(KPR) TASP peptide, a potent and selective ligand of cell surface nucleolin, was synthesized by the solid phase method using protected Fmoc amino acids and Wang resin as the solid support [16]. In summary, Fmoc-protected amino acids were used with the t-Butyl group as side-chain protecting group for Glu, tert-butuloxycarbony group (t-Boc) and/or 4-methoxytrityl group for Lys, t-Boc group for Trp, trityl group for Cys and 2,2,4,6,7-pentamethyldihydrobenzofuran-5-sulfonyl group for Arg. Stepwise synthesis of the TASP core peptide was achieved using diisopropylcarbodiimide/1-hydroxybenzotriazole as coupling reagents. The KPR tripeptide was step by step synthesized on ε-aminogroup of the lysine residues in positions 1,3,6,8 as well on the aminoterminal of the TASP core using HATU/DIPEA in dimethylformamide as coupling agents. After completion of the synthesis, the resin was treated with a trifluoroacetic acid solution (TFA/1,2-ethanedithiol/triethylsilane/water/anisole, 95/1/1/1/2 v/v/v/v/v) in the presence of scavengers to liberate the fully deprotected crude peptide. The released peptide was precipitated upon solvent concentration and addition of cold ether and the final product was purified by gel filtration chromatography on Sephadex G-15 using 20% acetic acid as the eluent. Final purification was achieved by preparative high performance liquid chromatography. The final 5(KPR)TASP peptide was checked for its purity by analytical HPLC on a Lichrosorb RP18 column (C18 solid phase, 7 μm particle size, 250 mm × 8 mm) applying a linear gradient 10%-70% acetonitrile (0.1% TFA) for 35 minutes, and 70%- 100% acetonitrile (0.1% TFA) for 5 minutes (flow rate 1.5 mL/min, UV detection at 220 nm and 254 nm). The final verification of the peptide sequence was achieved by Electron Spray Ionization-Mass Spectrometry.

### Statistical analysis

The significance of variability between the results from various groups was determined by one way ANOVA. Each experiment included triplicate measurements for each condition tested, unless otherwise indicated. All results are expressed as mean ± S.E.M. from at least three independent experiments.

## Results

### PTN is expressed by chicken embryo CAM cells and interacts with RPTPβ/ζ

**Figure 1:**
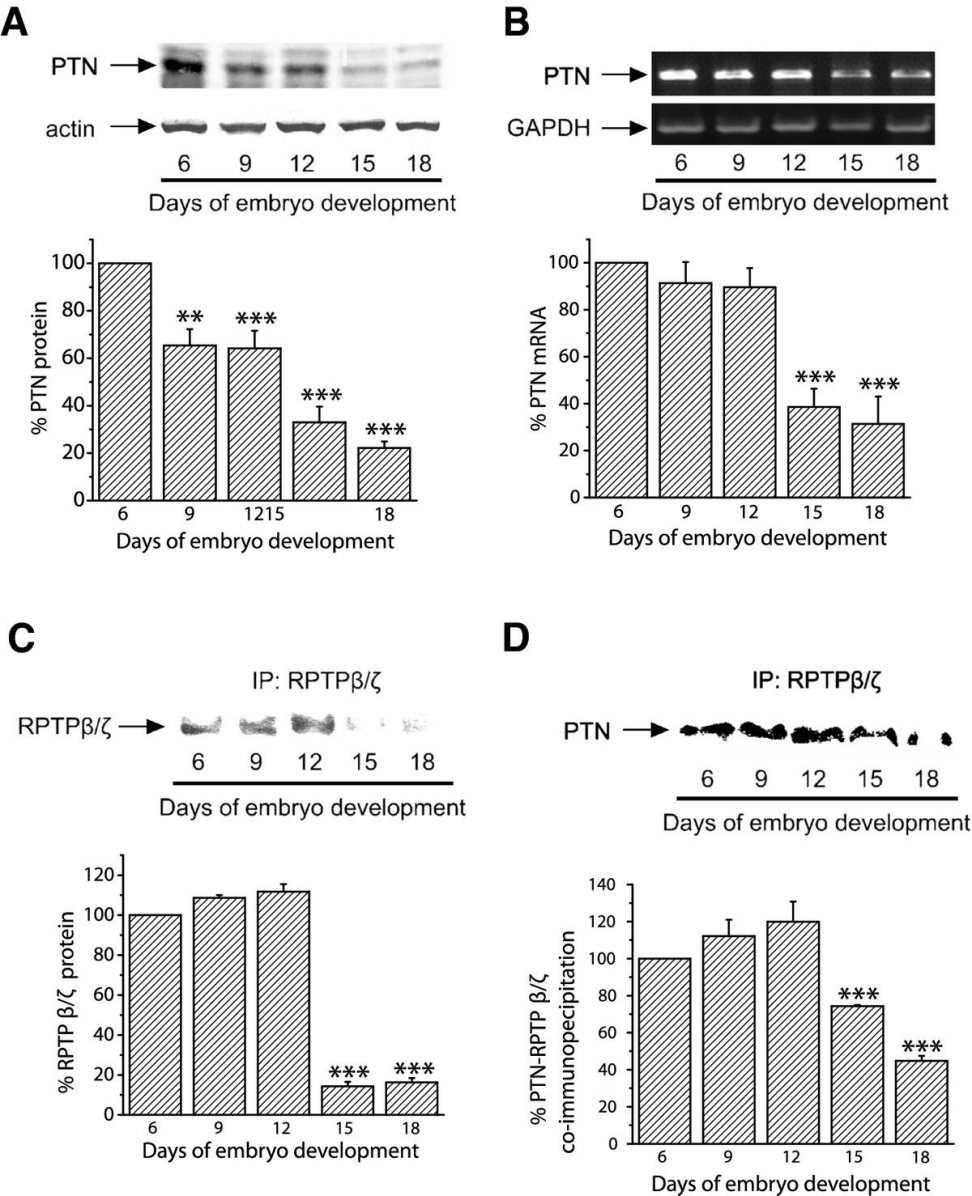
**Detection of endogenous PTN and RPTPβ/ζ in the chicken embryo CAM**. **A**. Equal amounts of protein extracts of chicken embryo CAM from different developmental stages were analyzed by SDS-PAGE, followed by Western blot analysis for PTN and actin. The protein amounts were quantified by densitometric analysis of the corresponding bands and the ratio PTN/actin was calculated for each lane. **B**. Products of RT-PCR reactions for chicken PTN and GAPDH from mRNA of chicken embryo CAM from different developmental stages. The mRNA amounts were quantified by densitometric analysis of the corresponding bands and the ratio PTN/GAPDH was calculated in each lane. Results in both A and B are expressed as mean ± S.E.M. of the % change of the PTN relative amounts compared to day 6. **C**. Three mg of total protein from chicken embryo CAM extracts from different developmental stages were subjected to immunoprecipitation for RPTPβ/ζ. Precipitated proteins were analyzed by SDS-PAGE, followed by Western blot for the presence of RPTPβ/ζ. **D**. Three mg of total protein from chicken embryo CAM extracts from different developmental stages were subjected to immunoprecipitation for RPTPβ/ζ. Precipitated proteins were analyzed by SDS-PAGE, followed by Western blot analysis for the presence of PTN. The protein amounts in C and D were quantified by densitometric analysis of the corresponding bands and results are expressed as mean ± S.E.M. of the % change compared to day 6. Asterisks in all cases denote a statistically significant difference from day 6. **P < 0.01, ***P < 0.001.

Initially, we investigated whether PTN is physiologically expressed by CAM cells. In order to do this, we performed Western blot and RT-PCR analyses to detect PTN at several stages of embryo development. Equal amounts of total CAM proteins from tissues of different developmental stages were analyzed by 17.5% SDS-PAGE. Western blot analysis showed that PTN protein was present in the chicken embryo CAM at all embryonic developmental stages, its amounts were maximal at day 6 of embryo development and declined progressively until day 18 (Figure 1A). Using semi-quantitative RT-PCR, it was found that PTN mRNA levels were also maximal at day 6 of embryo development, remained at high levels till day 12 and were decreased at later developmental stages (Figure 1B). RPTPβ/ζ protein was also present in the chicken embryo CAM at all embryonic developmental stages, its amounts were maximal at days 6-12 of embryo development and declined progressively until day 18 (Figure 1C). We could not detect RPTPβ/ζ at the mRNA level since the chicken gene is not known and the human primers tested did not form any product. PTN and RPTPβ/ζ co-immunoprecipitated from CAM protein extracts and this interaction was also maximal at days 6-12 of embryo development (Figure 1D). Interestingly, integrin α_ν_β_3 _also interacts with RPTPβ/ζ when there is active angiogenesis, while this interaction is decreased when angiogenesis of the tissue has stopped (Additional file 1).

### Decrease of endogenous PTN expression results in decreased angiogenesis in the chicken embryo CAM

**Figure 2:**
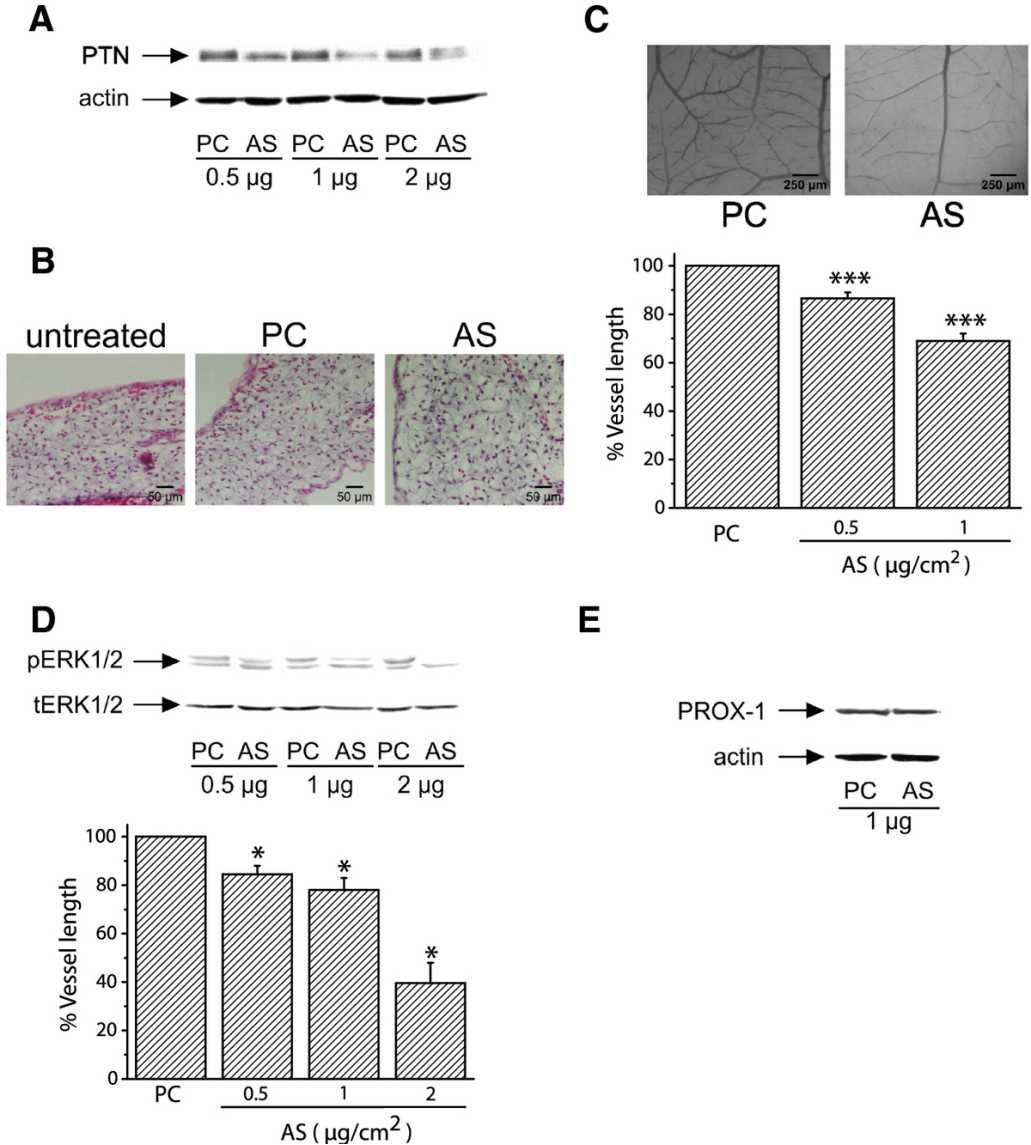
**Decreased expression of endogenous PTN following transfection of CAM cells with pCDNA3.1 carrying AS-PTN results in decreased angiogenesis**. **A**. Equal amounts of protein extracts of chicken embryo CAM 24 h after application of pCDNA3.1 alone (PC) or carrying AS-PTN (AS) were analyzed by SDS-PAGE, followed by Western blot analysis for PTN and actin. **B**. Haematoxylin & eosin staining of CAM paraffin sections 24 h after application of 1 μg of pCDNA3.1 alone (PC) or carrying AS-PTN (AS). Untreated CAM paraffin sections at the same developmental stage were used as control. **C**. Different amounts of pCDNA3.1 alone or carrying AS-PTN were applied on the CAM and 48 h later the number of vessels was estimated using image analysis software. Results are expressed as mean ± S.E.M. of the % change of the total vessel length in treated with pCDNA3.1 carrying AS-PTN (AS) compared with the tissue treated with pCDNA3.1 vector alone (PC) at the same quantity (control). Asterisks denote a statistically significant difference (unpaired *t*-test) from the control. ***P < 0.001. The pictures are representative, showing the vessel network of the chicken embryo CAM after treatment with 1 μg pCDNA3.1 alone (PC) or carrying AS-PTN (AS). **D**. Representative pictures of Western blot analyses from three independent experiments for phosphorylated ERK1/2 (pERK1/2) and total ERK1/2 (tERK1/2) in protein extracts of chicken embryo CAM 24 h after application of pCDNA3.1 alone (PC) or carrying AS-PTN (AS). Phospho- and total protein amounts were quantified by densitometric analysis of the corresponding band in each lane, and the ratio pERK1/2/tERK1/2 was calculated in each lane. Results are mean ± S.E.M. of the % change of ERK1/2 phosphorylation in treated with pCDNA3.1 carrying AS-PTN (AS) compared with the tissue treated with pCDNA3.1 vector alone (PC) at the same quantity (control). Asterisks denote a statistically significant difference (unpaired *t*-test) from the control. *P < 0.05. **E**. Representative pictures of Western blot analyses from three independent experiments for PROX-1 and actin in protein extracts of chicken embryo CAM 24 h after application of pCDNA3.1 alone (PC) or carrying AS-PTN (AS).

Since PTN expression correlates with blood vessel formation in the CAM, we studied if down-regulation of its expression would decrease angiogenesis of the tissue. AS-PTN or empty pCDNA3.1 vectors were applied on the CAM at day 9 of embryo development, as described in Methods. As shown in Figure 2A, application of AS-PTN onto CAM led to decreased amounts of endogenous PTN in a dose-dependent manner, suggesting that expression of PTN was significantly affected by this treatment, without being toxic to the tissue as evidenced by haematoxylin-eosin staining of CAM paraffin sections (Figure 2B). Decreased expression of endogenous PTN by CAM cells correlated with a dose-dependent decrease in tissue angiogenesis (Figure 2C) and ERK1/2 activity (Figure 2D). In contrast, it did not affect the expression of the lymphatic endothelial cells' marker PROX-1 (Figure 2E). Similarly to CAM angiogenesis, PROX-1 expression is increased till day 12 of embryo development and significantly decreased at later developmental stages (Additional file 2).

### PTN directly interacts with NCL in CAM and human endothelial cells

**Figure 3:**
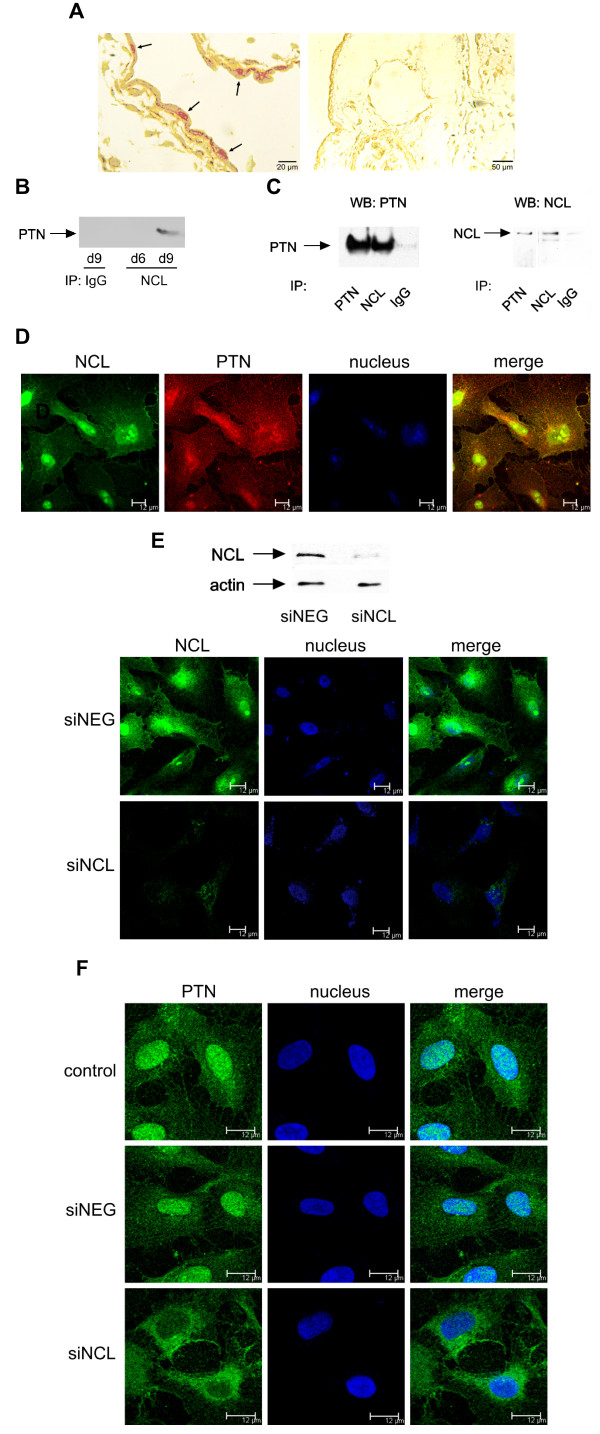
**NCL is playing a role in the nuclear translocation of PTN**. **A**. Cellular localization of PTN protein in paraffin sections of chicken embryo CAM at day 9 of embryo development. Arrows on the left picture indicate an intense PTN immunoreactivity in the nucleus of endothelial cells (40× magnification). The picture on the right shows a CAM paraffin section treated only with the secondary antibody (20× magnification). **B**. Equal amounts of protein extracts of chicken embryo CAM from days 6 (d6) and 9 (d9) of embryo development were subjected to immunoprecipitation for IgG or NCL. The precipitated proteins were analyzed by Western blot for the presence of PTN. **C**. Equal amounts of protein extracts of HUVEC were subjected to immunoprecipitation for IgG, PTN or NCL. The precipitated proteins were analyzed by SDS-PAGE, followed by Western blot analysis for the presence of NCL or PTN respectively. **D**. Representative immunofluorescent images of HUVEC stained for PTN (red), NCL (green) and nucleus (blue). **E**. HUVEC lysates or fixed cells after treatment for 24 h with siRNA for NCL were analyzed for the presence of NCL, by Western blot or immunofluorescence respectively. **F**. Representative immunofluorescent images of HUVEC stained for PTN (green) and nucleus (blue) after down-regulation of NCL expression by siRNA. siNEG, cells transfected with a negative control siRNA; siNCL, cells transfected with siRNA for NCL.

By using immunohistochemistry in CAM paraffin sections we found that endothelial cells were positive for PTN; it is noteworthy that PTN immunoreactivity was also detected in the nucleus of CAM endothelial cells (Figure 3A). Based on this observation, we studied whether PTN interacts with NCL, a protein that participates in the transport between cytoplasm and nucleus of several molecules [17-19], among which midkine [10]. By immunoprecipitating CAM proteins against NCL and analyzing immunoprecipitates for the presence of PTN, we found that PTN co-immunoprecipitated with NCL (Figure 3B). We verified PTN nuclear localization, as well as PTN-NCL interaction in HUVEC, by a combination of immunoprecipitation, Western blot and immunofluorescent analyses. PTN was co-immunoprecipitated with NCL in HUVEC lysates (Figure 3C) and co-localized with NCL in the nucleus, cytoplasm and cell membrane of HUVEC (Figure 3D). By performing subcellular fractionation assay, similar pattern of co-localization of PTN with NCL was also observed in U87MG cancer cells (Additional file 3), suggesting that the interaction between PTN and NCL is common to several types of cells. In order to investigate whether NCL is playing a role in the nuclear localization of PTN, we down-regulated NCL expression in HUVEC by siRNA (Figure 3E), and examined the subcellular localization of PTN by immunofluorescence microscopy. As shown in Figure 3F, when NCL expression was down-regulated, PTN localization in the nucleus was significantly decreased.

### Down-regulation of NCL abolishes PTN-induced migration of human endothelial cells

**Figure 4:**
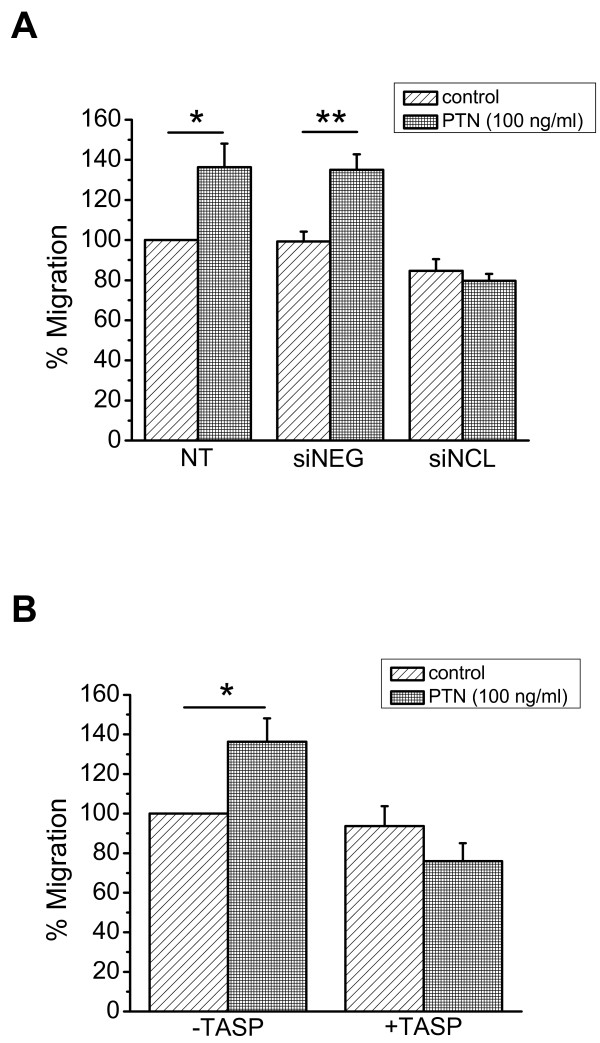
**NCL plays a role in PTN-induced endothelial cell migration**. **A**. Down-regulation of NCL expression by siRNA completely abolished PTN-induced HUVEC migration. **B**. Blockage of cell surface NCL by its ligand 5(KPR)TASP (TASP, 1 μM) completely abolished PTN-induced HUVEC migration. Results are expressed as percent of the values obtained in untreated cells (control). Data are the mean ± S.E.M. of three independent experiments. Asterisks denote a statistically significant difference from control. *P < 0.05, **P < 0.01. NT, non transfected cells; siNEG, cells transfected with a negative control siRNA; siNCL, cells transfected with siRNA for NCL.

In order to determine whether NCL is involved in PTN-induced endothelial cell migration, we studied PTN-induced migration of HUVEC after down-regulation of NCL expression by siRNA. As shown in Figure 4A, down-regulation of NCL expression resulted in complete inhibition of PTN-induced endothelial cell migration. We furthermore studied PTN-induced migration of HUVEC in the presence of the 5(KPR)TASP pseudopeptide that is known to bind the C-terminal RGG domain of cell-surface expressed NCL and block its function [16]. 5(KPR)TASP pseudopeptide completely abolished PTN-induced HUVEC migration (Figure 4B), further suggesting involvement of NCL in the angiogenic functions of PTN. The concentration of PTN used in all *in vitro *assays was 100 ng/ml, which causes a maximal effect on endothelial cell migration [4].

## Discussion

PTN is expressed in a variety of primary human tumours and has been correlated with enhanced tumour growth and vascular density reviewed in [2]. We have previously shown that exogenously administered human recombinant PTN is angiogenic in several *in vitro *models and in the chicken embryo CAM model *in vivo *[3]. In the present study we show that endogenous PTN is playing a significant role in the vascularisation of the chicken embryo CAM. This is supported by our findings that: a) Expression of endogenous PTN is maximal at early developmental stages, when there is active endothelial cell proliferation, migration and angiogenesis, and is decreased later, when there is no endothelial cell proliferation and migration [20], and b) decreased expression of endogenous PTN resulted in decreased angiogenesis of the tissue, directly linking PTN expression with new blood vessel formation. In favour of a stimulatory effect of PTN on *in vivo *angiogenesis are also our additional data showing that exogenously added PTN onto the chicken embryo CAM increases the mRNA levels of vascular endothelial growth factor (VEGF) isoforms 165 and 190, as well as activates metalloproteinase 2 (Additional file 4). These data are in line with a recent work showing that PTN promotes VEGF expression and cooperates with VEGF in promoting colorectal cancer angiogenesis [21]. Interestingly, although PTN seems to affect the number of angiogenic blood vessels, it did not affect lymphatic endothelial cells' marker PROX-1 expression, suggesting that PTN does not affect lymphatic vessel density, in line with data on colorectal cancer showing that lymphatic microvessel density does not correlate with PTN expression [21].

The mechanisms involved in the angiogenic action of PTN *in vivo *are not completely clear. We have previously shown that RPTPβ/ζ is responsible for PTN-induced endothelial cell migration [4]. In the chicken embryo CAM, we have detected RPTPβ/ζ at the protein level and found that it interacts with PTN and its expression is maximal at the early developmental stages, similarly to the expression of PTN. Besides RPTRPβ/ζ, α_ν_β_3 _integrin seems to be critical for the stimulatory effect of PTN on endothelial cell migration, by forming a functional complex with RPTPβ/ζ on the cell surface [5]. Integrin α_ν_β_3 _directly interacts with PTN and RPTPβ/ζ in the chicken embryo CAM [5], is activated on CAM endothelial cells only when there is active angiogenesis [7], and interacts with RPTPβ/ζ when there is active angiogenesis, while this interaction is decreased when angiogenesis of the tissue has stopped. However, besides a direct effect on endothelial cells, it is also possible that PTN has an indirect effect, potentiating the angiogenic effect of other growth factors. For example, it has been previously shown that PTN induces proliferation of human peripheral blood mononuclear cells [22] and increases the mRNA expression of the VEGF receptor Flt-1 in endothelial cell cultures [23]. Flt-1 is expressed by monocytes and its expression is increased after monocyte activation [24]. In the same line, recent findings show that PTN induces transformation of monocytes into functional endothelial cells, thus supporting angiogenesis [25,26]. It may be possible that at least part of the angiogenic effect of PTN in the chicken embryo CAM is due to activation of CAM blood cells.

It is well known that PTN activates ERK1/2 in several types of cells [27-29], among which endothelial cells [4], and this pathway has been linked to stimulation of endothelial cell proliferation [28] and migration [4]. In the present study we show that decreased expression of endogenous PTN leads to decreased ERK1/2 activity, which correlates with the effect on tissue angiogenesis, suggesting that PTN-induced ERK1/2 activation may be important for the stimulation of PTN's angiogenic activities *in vivo*, similarly to what has been known from *in vitro *studies [4,28].

It is noteworthy that in the CAM, endogenous PTN is also localized in the nucleus of endothelial cells. Similar observation has been obtained using HUVEC and U87MG cells (present study), as well as other types of cells that express endogenous PTN, such as neonatal cardiomyocytes [30]. Over the past years, evidence has accumulated that several growth factors related to angiogenesis and tumour growth, as well as their receptors, are found in the cell nucleus. Although this nuclear distribution might be related to the transcriptional activation of genes involved in the angiogenic cascade, at the moment the precise function of this process is completely unknown (reviewed in [31]). Nuclear translocation of PTN has been previously discussed, based on the observations that its primary structure contains three potential nuclear targeting sequences [32], it was found to bind to NCL [33], and NCL was found to participate in midkine nuclear translocation [10,33]. Although interaction of PTN with NCL has been previously mentioned [8], it is the first time that it is clearly shown that NCL participates in PTN nuclear translocation. At the moment, the functional significance of the nuclear localization of PTN is completely unknown. One possibility for the role of nuclear PTN could be an involvement in cell cycle progression. It has been previously shown that interruption of PTN expression induces tetraploidy and aneuploidy in U87MG cells and may contribute to the reversal of their highly malignant phenotype [34]. Another possibility might be a role in apoptosis. There are many studies supporting an anti-apoptotic effect of PTN [35-38], in line with studies supporting an anti-apoptotic effect of NCL [39-41]. However, it has not been clear whether this anti-apoptotic effect is due to the nuclear localization of PTN and further studies are required to elucidate this point. Finally, it has been recently shown that NCL interacts with the G-rich strands in the pPu/pPy tract of the VEGF promoter and acts as transcriptional activator of the VEGF gene [42]. PTN also promotes VEGF expression [21] and the possibility that PTN and NCL co-operate in regulating expression of several molecules is interesting and is further studied.

Besides translocation to the nucleus, NCL seems to also participate in PTN-induced endothelial cell migration. This seems to be mediated mainly by the cell surface NCL and to the best of our knowledge, this is the first study to show direct interaction between the two molecules on the cell surface and implication of NCL in angiogenic functions of PTN. Extranuclear distribution of NCL has been observed in both endothelial [17] and cancer [19,43,44] cells and seems to participate in functions that lead to angiogenesis and tumour growth, through yet unknown mechanisms. A specific antagonist that binds the C-terminal tail of NCL, similarly to the 5(KPR)TASP peptide we used in the present study, decreased blood vessel branching in the chicken embryo CAM [43]. This peptide forms a stable complex with cell surface-expressed NCL [43], suggesting that the latter plays a role in CAM angiogenesis and participates in PTN-induced endothelial cell migration. A number of recent studies suggest that several molecules that affect angiogenesis and tumour growth act through cell surface NCL, such as hepatocyte growth factor [45], VEGF [46], endostatin [47,48] and tumour necrosis factor-alpha inducing protein [49], favouring the notion that targeting of cell surface NCL may prove to be an effective anticancer therapy [50,51].

In summary, our data suggest that expression of endogenous PTN and its receptor RPTPβ/ζ in the CAM is related to new blood vessel formation. PTN seems to be involved in angiogenesis of the tissue, possibly through interaction with its cell surface receptors and ERK1/2 activation. NCL interacts with PTN on the cell surface, is involved in the nuclear translocation of PTN and may have a role in the angiogenic functions of PTN both *in vivo *and *in vitro*.

## Competing interests

The authors declare that they have no competing interests.

## Authors' contributions

GD performed CAM transfections, Western blot analyses and angiogenesis assays following transfections. MK performed all migration assays, siRNA cell transfections, immunofluorescent assays and cell fractionations, participated in the immunoprecipitation assays and helped preparing the draft of the manuscript. CM, KT and DV participated in histochemistry, immunoprecipitation and Western blot assays on CAM tissues. PK participated in Western blot and immunohistochemistry assays at different developmental stages. EG performed immunohistochemistry and RT-PCR assays. JC participated in the design of the work related to the role of NCL. CP, VM and PC worked on the 5(KPR)TASP peptide synthesis. EP designed the work, performed the statistical analysis and wrote the manuscript. All authors read and approved the final manuscript.

## Supplementary Material

Additional file 1 **Co-immunoprecipitation of RPTPβ/ζ with α_ν_β_3 _in the chicken embryo CAM during embryo development**. Three mg of total protein from chicken embryo CAM extracts from different developmental stages were subjected to immunoprecipitation for α_ν_β_3_. Precipitated proteins were analyzed by SDS-PAGE, followed by Western blot analysis for the presence of RPTPβ/ζ. Both transmembrane splice variants of RPTPβ/ζ (Garwood *et al., J Biol Chem *2003, **278:**24164-24173) have been detected. Click here for file

Additional file 2 **Detection of PROX-1 in the chicken embryo CAM during embryo development**. Equal amounts of protein extracts of chicken embryo CAM from different developmental stages were analyzed by SDS-PAGE, followed by Western blot analysis for PROX-1 and actin. Click here for file

Additional file 3 **Interaction of PTN with NCL in human glioma U87MG cells**. **A**. Equal total protein amounts of subcellular fractions of U87MG cells were immunoprecipitated for NCL and PTN and analyzed by Western Blot analysis for the same molecules. **B **Equal total protein amounts of subcellular fractions of U87MG cells were immunoprecipitated for NCL or PTN and analyzed by Western Blot analysis for PTN or NCL respectively. It should be noted that nuclear NCL was also detected as a band of lower molecular mass than extranuclear NCL, in line with the described posttranslational modifications of extranuclear NCL with complex N- and Oglycosylations (Carpentier *et al., Biochemistry *2005, **44**:5804-5815). Click here for file

Additional file 4 **Exogenous administration of human recombinant PTN increased the mRNA levels of VEGF_190 _and VEGF_165 _and activated metalloproteinase (MMP)-2 in the chicken embryo CAM**. **A**. Products of RT-PCR reactions for chicken VEGF190 and VEGF165 and GAPDH from mRNA of chicken embryo CAM after application of different doses of PTN. The primers used in the present study amplified both variants of avian VEGF, as previously described (Giannopoulou *et al., J Pharmacol Exp Ther *2003, **304**:729-737). **B**. Equal amounts of total protein extracts of chicken embryo CAM from different developmental stages were analyzed by SDS-PAGE, followed by zymography for MMP-2 and Western analysis for actin. MMP-2 is the predominant metalloprotease detected in the CAM (Ribatti *et al., J Pharmacol Exp Ther *2003, **304**:729-737; Giannopoulou *et al., Int J Cancer *2001, **94**:690-698). Click here for file
