# Supplementary material for: Pleiotrophin expression and role in physiological angiogenesis in vivo: potential involvement of nucleolin
Source: Vasc Cell. 2012 Mar 16;4:4. doi: 10.1186/2045-824X-4-4 (PMC3379939; doi:10.1186/2045-824X-4-4)
Supplement: Additional file 1 — Co-immunoprecipitation of RPTPβ/ζ with ανβ3 in the chicken embryo CAM during embryo development. Three mg of total protein from chicken embryo CAM extracts from different developmental stages were subjected to immunoprecipitation for ανβ3. Precipitated proteins were analyzed by SDS-PAGE, followed by Western blot analysis for the presence of RPTPβ/ζ. Both transmembrane splice variants of RPTPβ/ζ (Garwood et al., J Biol Chem 2003, 278:24164-24173) have been detected. [file 2045-824X-4-4-S1.PDF]

Additional file 1

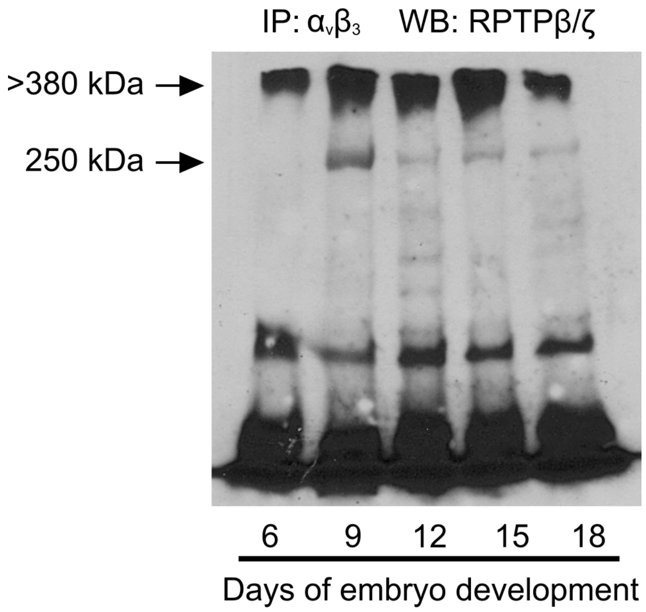

Co-immunoprecipitation of RPTPβ/ζ with $\alpha_v\beta_3$ in the chicken embryo CAM during embryo development. Three mg of total protein from chicken embryo CAM extracts from different developmental stages were subjected to immunoprecipitation for $\alpha_v\beta_3$. Precipitated proteins were analyzed by SDS-PAGE, followed by Western blot analysis for the presence of RPTPβ/ζ. Both transmembrane splice variants of RPTPβ/ζ (Garwood *et al.*, 2003) have been detected.

Garwood J, Heck N, Reichardt F, Faissner A: **Phosphacan short isoform, a novel non-proteoglycan variant of phosphacan/receptor protein tyrosine phosphatase-beta, interacts with neuronal receptors and promotes neurite outgrowth.** *J Biol Chem* 2003*,* **278:** 24164–24173.
