# Supplementary material for: Pleiotrophin expression and role in physiological angiogenesis in vivo: potential involvement of nucleolin
Source: Vasc Cell. 2012 Mar 16;4:4. doi: 10.1186/2045-824X-4-4 (PMC3379939; doi:10.1186/2045-824X-4-4)
Supplement: Additional file 2 — Detection of PROX-1 in the chicken embryo CAM during embryo development. Equal amounts of protein extracts of chicken embryo CAM from different developmental stages were analyzed by SDS-PAGE, followed by Western blot analysis for PROX-1 and actin. [file 2045-824X-4-4-S2.PDF]

Additional file 2

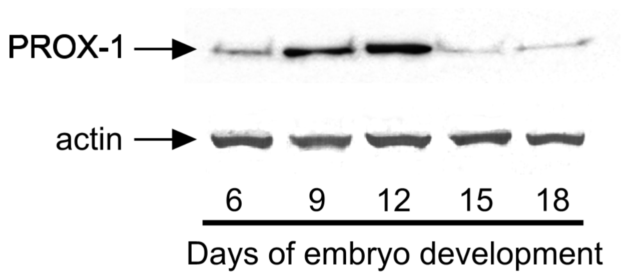

Detection of PROX-1 in the chicken embryo CAM during embryo development. Equal amounts of protein extracts of chicken embryo CAM from different developmental stages were analyzed by SDS-PAGE, followed by Western blot analysis for PROX-1 and actin.
