# Supplementary material for: Pleiotrophin expression and role in physiological angiogenesis in vivo: potential involvement of nucleolin
Source: Vasc Cell. 2012 Mar 16;4:4. doi: 10.1186/2045-824X-4-4 (PMC3379939; doi:10.1186/2045-824X-4-4)
Supplement: Additional file 3 — Interaction of PTN with NCL in human glioma U87MG cells. A. Equal total protein amounts of subcellular fractions of U87MG cells were immunoprecipitated for NCL and PTN and analyzed by Western Blot analysis for the same molecules. B Equal total protein amounts of subcellular fractions of U87MG cells were immunoprecipitated for NCL or PTN and analyzed by Western Blot analysis for PTN or NCL respectively. It should be noted that nuclear NCL was also detected as a band of lower molecular mass than extranuclear NCL, in line with the described posttranslational modifications of extranuclear NCL with complex N- and Oglycosylations (Carpentier et al., Biochemistry 2005, 44:5804-5815). [file 2045-824X-4-4-S3.PDF]

Additional file 3

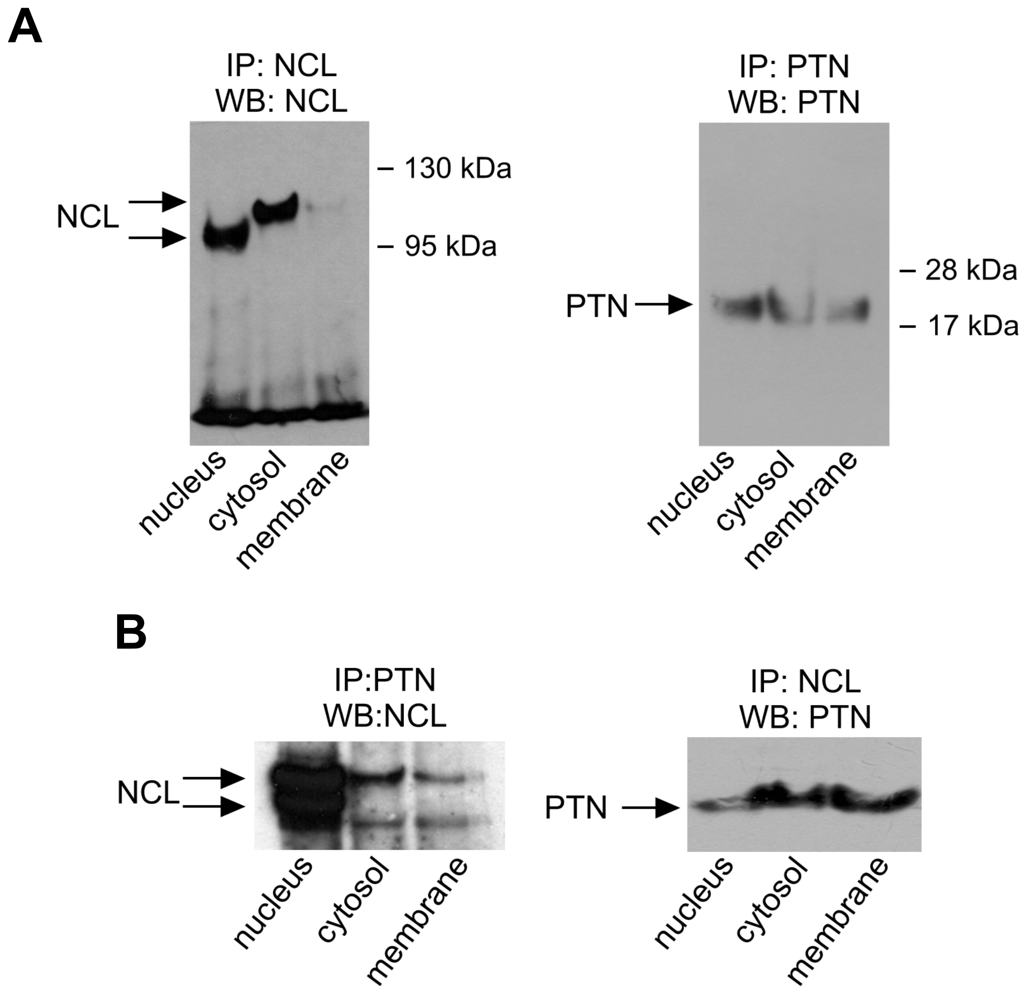

Interaction of PTN with NCL in human glioma U87MG cells. A. Equal total protein amounts of subcellular fractions of U87MG cells were immunoprecipitated for NCL and PTN and analyzed by Western Blot analysis for the same molecules. **B** Equal total protein amounts of subcellular fractions of U87MG cells were immunoprecipitated for NCL or PTN and analyzed by Western Blot analysis for PTN or NCL respectively. It should be noted that nuclear NCL was also detected as a band of lower molecular mass than extranuclear NCL, in line with the described posttranslational modifications of extranuclear NCL with complex N- and O-glycosylations (Carpentier *et al.*, 2005).

Carpentier M, Morelle W, Coddeville B, Pons A, Masson M, Mazurier J, Legrand D: **Nucleolin undergoes partial N- and O-glycosylations in the extranuclear cell compartment.** *Biochemistry* 2005, **44**: 5804-5815.
