# Supplementary material for: Pleiotrophin expression and role in physiological angiogenesis in vivo: potential involvement of nucleolin
Source: Vasc Cell. 2012 Mar 16;4:4. doi: 10.1186/2045-824X-4-4 (PMC3379939; doi:10.1186/2045-824X-4-4)
Supplement: Additional file 4 — Exogenous administration of human recombinant PTN increased the mRNA levels of VEGF190 and VEGF165 and activated metalloproteinase (MMP)-2 in the chicken embryo CAM. A. Products of RT-PCR reactions for chicken VEGF190 and VEGF165 and GAPDH from mRNA of chicken embryo CAM after application of different doses of PTN. The primers used in the present study amplified both variants of avian VEGF, as previously described (Giannopoulou et al., J Pharmacol Exp Ther 2003, 304:729-737). B. Equal amounts of total protein extracts of chicken embryo CAM from different developmental stages were analyzed by SDS-PAGE, followed by zymography for MMP-2 and Western analysis for actin. MMP-2 is the predominant metalloprotease detected in the CAM (Ribatti et al., J Pharmacol Exp Ther 2003, 304:729-737; Giannopoulou et al., Int J Cancer 2001, 94:690-698). [file 2045-824X-4-4-S4.PDF]

Additional file 4

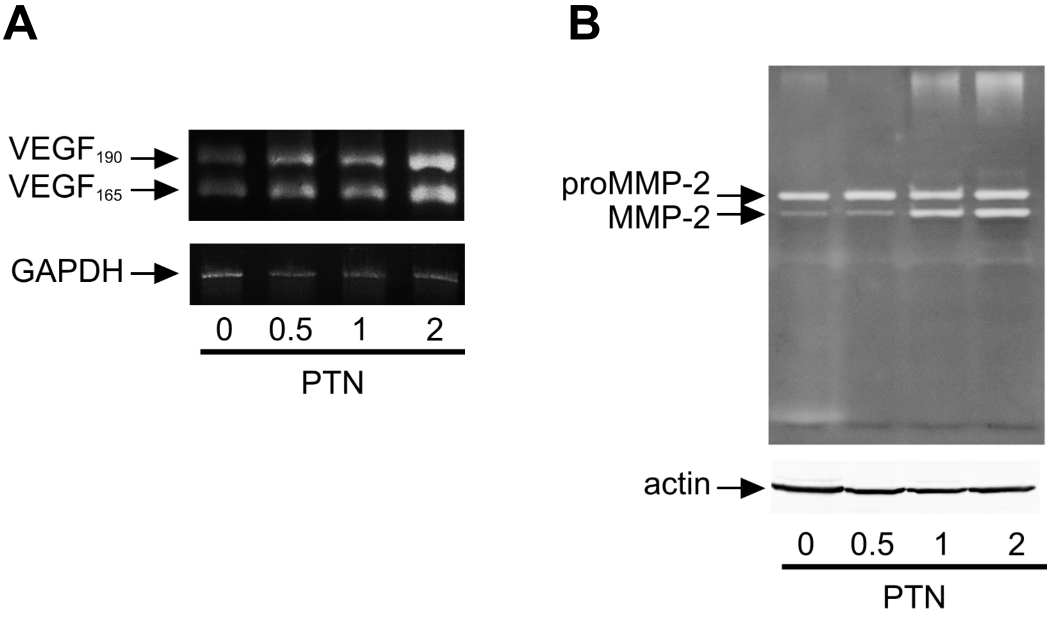

Exogenous administration of human recombinant PTN increased the mRNA levels of VEGF$_{190}$ and VEGF$_{165}$ and activated metalloproteinase (MMP)-2 in the chicken embryo CAM. A. Products of RT-PCR reactions for chicken VEGF$_{190}$ and VEGF$_{165}$ and GAPDH from mRNA of chicken embryo CAM after application of different doses of PTN. The primers used in the present study amplified both variants of avian VEGF, as previously described (Giannopoulou *et al.*, 2003). **B.** Equal amounts of total protein extracts of chicken embryo CAM from different developmental stages were analyzed by SDS-PAGE, followed by zymography for MMP-2 and Western analysis for actin. MMP-2 is the predominant metalloprotease detected in the CAM (Ribatti *et al.*, 1999; Giannopoulou *et al.*, 2001).

Giannopoulou E, Katsoris P, Hatziapostolou M, Kardamakis D, Kotsaki E, Polytarchou C, Parthymou A, Papaioannou S, Papadimitriou E. **X-rays modulate extracellular matrix in vivo.** *Int J Cancer* 2001, **94**: 690-698.

Giannopoulou E, Katsoris P, Kardamakis D, Papadimitriou E: **Amifostine inhibits angiogenesis in vivo.** *J Pharmacol Exp Ther* 2003, **304**: 729-737.

Ribatti D, Nico B, Vacca A, Iurlaro M, Roncali L: **Temporal expression of the matrix metalloproteinase MMP-2 correlates with fibronectin immunoreactivity during the development of the vascular system in the chick embryo chorioallantoic membrane.** *J Anat* 1999, **195**: 39-44.
